# A Hox-Embedded Long Noncoding RNA: Is It All Hot Air?

**DOI:** 10.1371/journal.pgen.1006485

**Published:** 2016-12-15

**Authors:** Licia Selleri, Marisa S. Bartolomei, Wendy A. Bickmore, Lin He, Lisa Stubbs, Wolf Reik, Gregory S. Barsh

**Affiliations:** 1 Program in Craniofacial Biology; Institute of Human Genetics; Eli and Edythe Broad Center of Regeneration Medicine & Stem Cell Research; Department of Orofacial Sciences & Department of Anatomy; University of California San Francisco, San Francisco, California, United States of America; 2 Department of Cell and Developmental Biology, University of Pennsylvania Perelman School of Medicine, Philadelphia, Pennsylvania, United States of America; 3 Medical Research Council Human Genetics Unit, Institute of Genetics and Molecular Medicine, University of Edinburgh, Edinburgh, United Kingdom; 4 Division of Cellular and Developmental Biology, University of California Berkeley, Berkeley, California, United States of America; 5 Institute for Genomic Biology, Department of Cell and Developmental Biology, University of Illinois, Urbana, Illinois, United States of America; 6 Department of Epigenetics, The Babraham Institute, Cambridge, United Kingdom; 7 HudsonAlpha Institute for Biotechnology, Huntsville, Alabama, United States of America; 8 Department of Genetics, Stanford University School of Medicine, Stanford, California, United States of America; The University of North Carolina at Chapel Hill, UNITED STATES

Over 20 years ago, the discovery of *Xist* as a critical component of X chromosome inactivation revealed a fundamental role for long noncoding RNAs (lncRNAs) in epigenetic regulation during mammalian development and foreshadowed a fascinating connection between RNA and chromatin modification [1–3]. In the last decade, the field has exploded, heralded in part by a 2007 landmark paper from the group of Howard Chang [4] describing that knockdown of a lncRNA (Hox Antisense Intergenic RNA [*HOTAIR*]) was associated with loss of transcriptional repression from a locus on another chromosome in trans. *HOTAIR* lncRNA—encoded within the *HOXC* locus, although its expression seemed to be required for normal epigenetic silencing of *HOXD* genes—became one of the most well-known examples of functional lncRNAs in the field of developmental epigenetics. Interest intensified when a subsequent paper from the Chang lab [5] reported that targeted deletion of the orthologous locus in the mouse (*Hotair*) caused homeotic transformations underpinned by derepression of *HoxD* gene transcription in vivo. Discovery of new lncRNAs and exploration of their potential actions and effects during development and disease is a continued source of excitement [6,7]. But questions about the effects and actions of *Hotair* have been controversial, raised in part by work from the group of Denis Duboule [8]. This debate is addressed directly in the current issue of *PLOS Genetics* in a manuscript from Duboule’s laboratory [9] that reanalyzes *Hotair* mutant mice generated by the Chang lab, a formal comment in response to that manuscript from the Chang lab [10], and this perspective.

A precise genomic organization conserved for hundreds of millions of years underlies the complexity of *Hox* gene regulation. Expression of adjacent *Hox* genes in specific spatiotemporal domains helps to pattern and maintain the identity of developmental compartments along the body and appendicular axes [8,11]. It is the latter part of that equation—maintenance of developmental identity—for which genomic organization may be so important, and for which epigenetic mechanisms play a critical role, because specific patterns of *Hox* gene transcription can be faithfully maintained long after development has concluded, sometimes in cultured cells outside the body [12].

These considerations, together with previous studies on the role of noncoding RNA (ncRNA) in the regulation of *Hox* gene expression in fruitflies [13], provided the backdrop for the work of Rinn et al. [4] to exhaustively profile noncoding (and coding) RNAs transcribed from the four human *HOX* loci. In flies, ncRNAs were shown to act in cis via transcriptional interference, so a potential trans-acting role for *HOTAIR*, from the *HOXC* to the *HOXD* cluster, came as something of a surprise.

It is in this context—a function for *HOTAIR*?—that the current work [9,10] pertains. The mouse *HoxC* cluster is ~140 kb in length; *Hotair* is a 2-exon transcript that lies in the region between *Hoxc11 and Hoxc12*. In the 2013 paper from Li et al., mice carrying an ~4 kb deletion that removed both *Hotair* exons variably exhibited a homeotic posterior transformation with five rather than six lumbar (L) vertebrae (L6 was transformed into the sacral (S)1 vertebra; L6→S1), as well as wrist bone malformations and subtle caudal anterior transformations [5]. The authors also found that *Hotair* RNA was enriched in RNA immunoprecipitates (RNA-IP) from E11.5 embryos using antibodies against components of the Polycomb repressive complex 2 (PRC2), which methylates histone H3 at lysine 27 (H3K27), or against the Lsd1 complex, which demethylates histone H3 at lysine 4 (H3K4) in vivo [5]. Finally, the authors reported that derepressed genes in *Hotair* mutant tail tip fibroblasts, including multiple members of the *HoxD* but not *HoxC* clusters, exhibited loss of H3K27me3 and gain of H3K4me3 [5]. Taken together, this work has been at the foundation of the viewpoint that *Hotair* functions as a trans-acting repressor of *HoxD* gene expression via recruitment of PRC2 [4,5].

But this viewpoint has been controversial. Part of the controversy stems from a 2011 study from the Duboule group [14] in which it was reported that E13.5 embryos or embryo fibroblasts carrying a complete deletion of the mouse *HoxC* locus exhibited no significant changes in *HoxD* expression or chromatin marks. At the time, this observation was interpreted as a difference in function between human *HOTAIR* and mouse *Hotair*. However, with publication of the 2013 paper from the Chang group [5], claiming a conserved function with regard to trans-acting repression of *HoxD* genes, there seemed to be a direct conflict between the two sets of observations [5,14].

How could the mice generated and studied by the Chang group, with a 4-kb deletion within the *HoxC* cluster, exhibit a more severe phenotype with respect to *HoxD* gene regulation than mice carrying a deletion of the entire *HoxC* cluster studied by the Duboule group? Li et al. [5] suggested that deletion of the entire cluster might have removed genes with functions that oppose that of *Hotair* and generously provided their animals (with the 4-kb deletion) to the Duboule group for additional analyses. The current issue of *PLOS Genetics* features the manuscript describing the results of this further investigation [9]. Although the analyses are extensive, the results are simple to summarize: Amandio et al. [9] report that, in their hands, the *Hotair* deletion allele generated by the Chang lab does not cause up-regulation of *HoxD* genes and associated homeotic transformations. Amandio et al. [9] do identify and confirm subtle anterior transformations in caudal vertebrae but suggest that this difference may be caused by changes in *HoxC* expression due to local effects of the deletion.

How can two groups reach essentially opposite conclusions studying the exact same allele? We consider this apparent paradox with regard to both the whole animal and the transcriptomic phenotypes. First, and as pointed out here in a formal comment from the Chang group [10], their 2013 study [5] was carried out on an inbred background (C57BL/6), whereas the Duboule group deliberately used a mixed background that includes both C57BL/6 and CBA genomes [9,14]. Potential advantages of an inbred background include the ability to compare mutant and nonmutant animals across space and time (although the Chang lab results are based on litters that carry both mutant and nonmutant animals), whereas advantages of a mixed background include the potential for inter-individual stabilization of variably expressed phenotypes due to canalization [15,16]. Indeed, the L6→S1 transformation causing 5 instead of 6 lumbar vertebrae (the most important phenotype from the perspective of evaluating the proposed effect of /*Hotair*/ on /*HoxD*/ derepression) is variably expressed. Li et al. reported that 18/31 mutant embryos had 5 lumbar vertebrae compared to 1/11 non-mutants [5]. By contrast, Amandio et al. [9] report that most of the embryos they examined, 8/10 mutant and 8/11 nonmutant, had 5 lumbar vertebrae. In other words, both mutant and nonmutant animals examined by the Duboule lab had the same phenotype described as characteristic for mutants in the Chang lab.

In fact, the boundary between lumbar and sacral vertebrae has been an oft-studied and intensive subject of investigation by mouse geneticists for decades and might best be described as capricious. The total number of lumbar vertebrae (5 or 6) and “sacralization” of L6 vary not only between inbred strains but also within the same strain and can be influenced by sex, maternal diet, age, parity, litter size, and environmental temperature [17–19]. Although these considerations do not change the statistical significance (*p* = 0.0002) of the observation by Li et al. [5], they do call into question its biological significance, as pointed out more than 50 years ago by Earl Green [17], Anne McLaren [20], and Hans Grüneberg [21].

What about the different transcriptomic conclusions? Li et al. [5] generated RNA-Seq data from duplicate cultures of tail tip fibroblasts (mutant, nonmutant, and heterozygous), whereas the RNA-Seq studies of Amandio et al. [9] were obtained from duplicate microdissections of six different E12.5 embryonic regions (forelimb, hindlimb, genital tubercle, and lumbosacral, sacrocaudal, and caudal trunk). Li et al. had made their raw data publicly available, allowing Amandio et al. to present and analyze RNA-Seq reads from both the tail tip fibroblasts and the embryonic fragments with the exact same bioinformatics pipeline, from alignment to annotation to inferences regarding differential expression. An example from the unique read count data is informative: in the study of Li et al., *Hoxd10* was the most highly expressed *HoxD* gene in tail tip fibroblasts, and there were 4,4,7,8,15, and 15 unique reads for duplicates from nonmutant, heterozygous, and mutant cells, respectively. In contrast, in the caudal embryonic tissue (the posterior-most trunk segment where *Hotair* is highly expressed), there were 1,654, 1,475, 1,289, and 1,315 unique *Hoxd10* reads, for duplicates from nonmutant and mutant tissues, respectively. These data represent a general theme of the transcriptome studies from both groups: the tail tip fibroblasts studied by Li et al. [5] express *HoxD* genes (and, incidentally, *Hotair*) at very low levels, whereas the embryonic tissue fragments studied by Amandio et al. [9] exhibit expression of *HoxD* (and *Hotair*) at much higher levels, allowing for statistically robust comparisons.

Additional studies in both papers [5,9] and additional *Hotair* alleles in other papers [22] raise additional questions. How could *Hotair* affect cells or embryonic regions such as the forelimb or lumbar trunk where, according to Amandio et al. (9), its expression was not detectable? What might account for a subtle anterior vertebral transformation (in the caudal region), a phenotype upon which both groups agree? This phenotype is typically caused by loss- rather than gain-of-function alterations in *Hox* genes. And to what extent are the phenotypes caused by the *Hotair* deletion due to *Hotair* itself or cis-regulatory alterations in *HoxC* gene expression? Regardless, neither these questions nor their potential answers provide much additional help in resolving the somewhat provocative query posed by Amandio et al: Is *Hotair* homeotic or homeopathic? Readers should reach their own conclusions based on the data [5,9,10], but our collective impression is that *Hotair* is not quite homeopathic, but it is also not a major determinant of developmental identity.

From the standpoint of mechanism, *Hotair* RNA was proposed to affect gene expression in *trans* at least in part by targeting PRC2 to genes. However, questions also remain about the potential biological significance of the *Hotair*-PRC2 association. This is in part because *Polycomb* target genes identified by Li et al. [5] in tail tip fibroblasts (by H3K27me ChIP-seq) did not significantly overlap with *Hotair* target genes identified by Amandio et al. in vivo [9]. Although several labs have observed and/or questioned how PRC2 interacts with *Hotair* RNA in vitro, an important challenge for the field is to understand what happens in vivo [23–25].

One of the reasons research in the lncRNA field has been both fruitful and controversial is the ability of exquisitely sensitive genome-scale technologies to identify transcripts expressed at very low levels, which is part of a more general debate about how to distinguish transcriptional noise from transcription with a biological function [26,27]. Going forward, we believe the developmental genetics community will best be served by studying lncRNAs and epigenetic modulators that yield robust and highly penetrant whole-animal phenotypes, and that may act by a variety of different mechanisms [6,7]. Fortunately, there are many from which to choose [22,28] and evolutionarily informed strategies to guide that choice [29].

We also wish to comment that the specific controversy itself is not as important as the ways in which it has been addressed. The Chang group made all of their data publicly available and provided the mouse they generated to the Duboule group. This should be the norm across the scientific community, but there are frequent exceptions, and the willingness of the Chang group to share their data and resources should be noted and appreciated by all concerned. Furthermore, the authors, reviewers, and editors of the Amandio et al. manuscript recognized the importance of publishing (largely) negative data, which, in this case, is likely to have a substantial impact on the field. Overall, transparency, open access principles, and collegiality are the champions in the debate about *Hotair*.
